# Case Report: CT manifestations of acute portal vein thrombosis: cases report and literature review

**DOI:** 10.3389/fradi.2025.1662089

**Published:** 2025-11-04

**Authors:** Lin Zhou, Zhi-cheng Huang, Xiao-hui Lin, Shao-jin Zhang, Ya He

**Affiliations:** ^1^ Department of Radiology, The 3rd Affiliated Hospital of Chengdu Medical College & Pidu District People’s Hospital, Chengdu, China; ^2^ Heart, Lung and Vessels Center, Sichuan Academy of Medical Sciences and Sichuan Provincial People’s Hospital, Chengdu, Sichuan, China

**Keywords:** acute portal vein thrombosis, CT, imaging findings, evaluation, treatment

## Abstract

Acute portal vein thrombosis (APVT) is a rare condition characterized by recent thrombus formation within the main portal vein or its branches. APVT occurring in patients without underlying cirrhosis or malignancy represents an even rarer presentation, with an estimated prevalence of 0.7–3.7 per 100,000 individuals. However, it can lead to severe complications, including intestinal infarction and mortality. We report two cases presenting with abdominal pain without an apparent precipitating factor. Both patients were diagnosed with APVT based on contrast-enhanced computed tomography (CT) findings, clinical presentation, and laboratory parameters. Depending on the extent of portal vein occlusion, distinct therapeutic approaches were employed: one patient underwent interventional therapy combining transjugular mechanical thrombectomy/thrombolysis with transjugular intrahepatic portosystemic shunt (TIPS) placement, while the other received systemic pharmacological thrombolysis. Successful portal vein recanalization was achieved in both patients, who subsequently recovered and were discharged. These cases underscore that prompt diagnosis and management of APVT can avert adverse clinical outcomes. Contrast-enhanced CT demonstrates significant value in classifying APVT, assessing disease severity, evaluating treatment response, and identifying complications, thereby providing crucial evidence for clinical decision-making.

## 1 Introduction

Acute portal vein thrombosis (APVT) refers to the formation of a thrombus in the main portal vein or its branches within the recent past (<30 days) (1). Portal vein thrombosis is a rare condition, with a global incidence rate ranging from 0.05%–0.5% in autopsy studies (2). The majority of cases occur in patients with underlying liver cirrhosis (3). In contrast, APVT occurring in patients without liver cirrhosis or malignancy is considerably rarer, with an estimated prevalence of approximately 0.7–3.7 per 100,000 individuals (4). APVT can lead to serious complications (5), including intestinal infarction and death. Clinical manifestations of APVT vary widely, ranging from asymptomatic presentation in cases of partial thrombosis to septicemia and hypotension in patients with complete occlusion. Contrast-enhanced computed tomography (CT) plays a vital role in assessing the classification, severity, treatment response, and complications associated with APVT.

Herein, we present two male patients diagnosed with APVT. We analyze their clinical characteristics and CT imaging findings. Following distinct treatment pathways—interventional radiology for one and systemic medical therapy for the other—both patients achieved portal vein recanalization and recovered.

## 2 Case presentation

### 2.1 Case 1

A 35-year-old male presented with upper abdominal pain of one week's duration, characterized as intermittent dull pain with episodic exacerbation, occurring without obvious inciting factors. The pain worsened after eating but was unaffected by positional changes. There was no radiation, referred pain, acid reflux, belching, hematemesis, or melena. Initial upper endoscopy revealed chronic atrophic gastritis (C1) with erosions and bile reflux; testing for Helicobacter pylori (HP) was positive. A diagnosis of chronic gastritis was made, and treatment with acid suppression, gastric mucosal protection, prokinetics, and HP eradication was initiated. However, the abdominal pain intensified three days later, prompting hospital admission. Physical examination revealed epigastric tenderness without rebound tenderness or palpable masses; Murphy's sign was negative. Laboratory findings included: D-dimer 3.32 mg/L, prothrombin time 21.1 s, fibrinogen 4.426 g/L; potassium 3.15 mmol/L, alanine aminotransferase (ALT) 168 U/L, aspartate aminotransferase (AST) 63 U/L, gamma-glutamyl transferase (GGT) 182 U/L, alkaline phosphatase (ALP) 167 U/L, total bile acids 27.0 μmol/L, blood glucose 9.55 mmol/L. On the third hospital day, the patient experienced worsening hemorrhoids. The patient was admitted for further evaluation. An upper abdominal Doppler ultrasound was performed on hospital day 2, which revealed hepatic steatosis but failed to identify the portal vein thrombosis. Consequently, a contrast-enhanced CT scan was obtained on hospital day 7, which confirmed the diagnosis.

#### 2.1.1 CT findings

**Figure 1:**
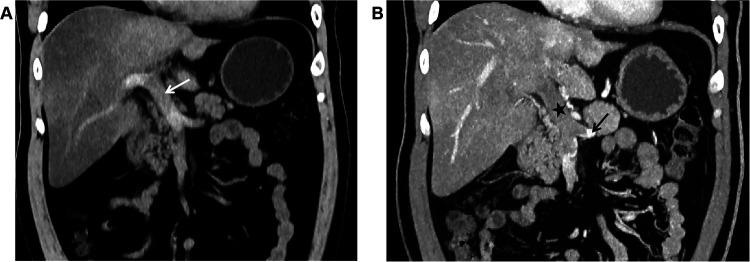
**(A)** In case 1, the non-contrast CT scan in the coronal plane of the abdomen shows widening of the main portal vein trunk (arrow), with unevenly increased density within the lumen, blurred margins of the vessel wall, and indistinct surrounding fat planes. **(B)** In Case 1, The CT scan with contrast during the portal venous phase in the coronal plane reveals filling defect changes in the main portal vein trunk (black star), along with the left branch and right branch, indicating complete occlusion of the lumen. The splenic vein (black arrow) is also involved, and no enhancement changes are noted in the filling defect area. The liver parenchyma exhibits reduced density, with significantly weakened enhancement post-contrast.

Unenhanced CT scan demonstrated dilation of the main portal vein trunk (diameter ∼1.89 cm) with heterogeneously increased intraluminal density (average CT value ∼45 HU, reaching up to 80 HU near the bifurcation), involving the left and right portal vein branches and the superior mesenteric vein (SMV) (Figure 1A). Portal venous phase clupontrast-enhanced CT revealed non-enhancing filling defects indicating complete occlusion of the main portal vein trunk, its left and right branches, the SMV, and the splenic vein (diameter ∼1.11 cm) (Figure 1B). Numerous tortuous, dilated collateral vessels with enhancement were noted periportally. The left gastric vein was dilated, draining into the left medial liver lobe; esophageal-gastric varices were not significantly dilated. Hypodensity was observed in the right lobe and lateral segment of the left lobe (CT value ∼30 HU), with significantly reduced enhancement compared to normal parenchyma. Bowel loops were normal in caliber without wall thickening, mesenteric stranding, obstruction, or abnormal enhancement. No ascites was present.

#### 2.1.2 Treatment and clinical outcomes

Due to extensive thrombosis resulting in complete occlusion of the main portal vein and its bilateral branches, a more aggressive interventional strategy beyond systemic anticoagulation was selected as first-line therapy. This decision was based on the following considerations: First, the duration from symptom onset to diagnosis was approximately two weeks, and unenhanced CT demonstrated hyperdensity within the portal vein thrombus (reaching up to 80 HU near the bifurcation), both of which indicated a subacute or early organized thrombus. Under such circumstances, the efficacy of anticoagulation therapy alone in achieving vascular recanalization is significantly reduced. Second, the extensive involvement of the thrombosis (complete occlusion of the main portal vein, its branches, and the splenic vein) implies a formidable risk of complications. Therefore, a combined regimen of transjugular mechanical thrombectomy/thrombolysis with TIPS placement was employed to actively restore portal venous flow and decompress the portal system, aiming to achieve immediate recanalization and prevent long-term complications. The patient was discharged on rivaroxaban. At one-year follow-up, the patient remained clinically stable. However, a recent follow-up abdominal CT revealed thrombus filling the TIPS stent and cavernous transformation of the portal vein at the hepatic hilum; liver function tests remained normal. Genetic testing identified a *PAI-1* 4G/5G heterozygous genotype, associated with increased thrombotic risk, leading to a diagnosis of hereditary thrombophilia.

### 2.2 Case 2

A 45-year-old male presented with continuous, distending abdominal pain of three days' duration, primarily localized periumbilically and in the upper abdomen, without apparent cause, radiation, or positional relief. The pain intensified one day prior to presentation. Symptomatic treatment at a community clinic provided no relief; he subsequently developed nausea and vomited gastric contents once, without hematemesis or coffee-ground material. Physical examination revealed tenderness in the right and lower abdomen with mild rebound tenderness, no significant guarding, no visible peristalsis, no costovertebral angle tenderness, negative Murphy's sign, and active bowel sounds. Laboratory results showed: D-dimer 7.32 mg/L, prothrombin time 15.7 s, fibrinogen 5.160 g/L; ALT 51 U/L, GGT 434 U/L, ALP 219 U/L, albumin 39.0 g/L, direct bilirubin 8.3 μmol/L. An abdominal contrast-enhanced CT scan was performed on the day of admission to establish the etiology of his acute abdomen.

#### 2.2.1 CT findings

**Figure 2:**
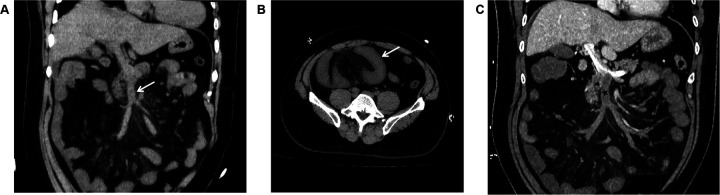
**(A)** In case 2, the non-contrast CT scan in the coronal plane of the abdomen displays enlargement of the superior mesenteric vein, with unevenly increased internal density, blurred vessel wall margins, and swelling of the surrounding mesentery (arrow). **(B)** In Case 2, The non-contrast CT scan in the axial plane shows thickening and swelling of the ileal wall, with increased density of the mucosal surface, and no dilation of the intestinal lumen. The serosal surface appears blurred, with associated mesenteric swelling, and fluffy shadows surrounding the blood vessels (arrow). **(C)** In Case 2, The CT scan with contrast during the portal venous phase in the coronal plane demonstrates filling defect changes within the superior mesenteric vein and the main portal vein trunk (black star). The superior mesenteric vein is completely occluded, and the main portal vein trunk is narrowed by approximately 50%, with no enhancement changes observed in the filling defect area. The thickened mucosal surface of the small intestine wall shows persistent enhancement, and a small amount of fluid is present around the liver.

Unenhanced CT scan demonstrated circumferential wall thickening (∼1.05 cm) predominantly involving ileal loops in the right lower abdomen and pelvis. The mucosal surface appeared hyperdense; luminal dilation or high-density intraluminal material was absent. The serosal surface was indistinct, with associated mesenteric swelling, perivascular haziness, and minimal fluid (Figures 2A,B). The SMV and main portal vein trunk were dilated (diameters ∼1.80 cm and ∼2.01 cm, respectively) with increased intraluminal density. Contrast-enhanced CT showed persistent enhancement of the thickened bowel wall mucosa. Filling defects were present within the SMV and main portal vein trunk: the SMV was completely occluded, while the main portal vein trunk showed approximately 50% stenosis; the filling defects showed no enhancement (Figure 2C). Liver parenchymal density and enhancement were unremarkable. A small amount of perihepatic fluid was noted.

#### 2.2.2 Treatment and clinical outcomes

Despite extensive SMV involvement, the main portal vein trunk was only partially occluded, and no significant ischemic changes were evident in the affected bowel. Despite extensive SMV involvement, the main portal vein trunk was only partially occluded, and no definitive signs of bowel necrosis (such as pneumatosis or portal venous gas) were evident. However, the presence of continuous abdominal pain with rebound tenderness, bowel wall thickening, mesenteric edema, and ascites raised concerns for impending bowel ischemia. Given the high risk of progression to intestinal infarction in the setting of complete SMV occlusion, systemic thrombolytic therapy was initiated to achieve rapid thrombus resolution and restore mesenteric venous flow, thereby averting bowel necrosis. Anticoagulation alone was deemed insufficient due to the severity of the clinical and radiological findings suggestive of early ischemia. Follow-up CT one week later demonstrated thrombus resolution. Despite thorough investigation, no underlying cause for the APVT (such as thrombophilia, malignancy, or local inflammatory conditions) was identified in this patient. Therefore, the APVT was considered idiopathic. Genetic testing for hereditary thrombophilic factors was not performed due to the patient's rapid clinical stabilization and favorable response to systemic thrombolytic therapy. Based on the absence of identifiable risk factors, this case was classified as idiopathic APVT. The patient remained well at one-year follow-up.

## 3 Discussion

### 3.1 Etiology and clinical aspects

APVT is defined by the recent formation of thrombi within the portal vein and other mesenteric veins, in the absence of pre-existing cavernous transformation or portal hypertension (6). Its pathogenesis aligns with Virchow's triad: venous stasis, endothelial injury, and hypercoagulability (7). Risk factors for APVT (6, 8) encompass acquired or hereditary thrombophilia, myeloproliferative neoplasms, paroxysmal nocturnal hemoglobinuria, systemic conditions (malignancy, autoimmune diseases, pregnancy), and local inflammatory or traumatic factors [appendicitis, diverticulitis (9), inflammatory bowel disease, pancreatitis, trauma, surgery]. Case 1 exemplifies hereditary thrombophilia associated with the *PAI-1* 4G/5G genotype. Type 1 plasminogen activator inhibitor (PAI-1) is the primary inhibitor of tissue-type plasminogen activator (tPA) and urokinase-type plasminogen activator (uPA) in the body's plasma, and it is responsible for regulating the balance between coagulation and fibrinolysis. Elevated levels of PAI-1 predispose the body to thrombus formation. Studies have shown that several polymorphisms in the PAI-1 gene are associated with the level of PAI-1 activity in plasma. It has been identified that a guanosine insertion/deletion polymorphism exists in the promoter region of the PAI-1 gene at the −675 bp position, and this polymorphism is referred to as the 4G/5G polymorphism. *in vitro* studies indicate that the 4G allele exhibits higher activity than the 5G allele; it can increase the plasma PAI-1 concentration by upregulating the expression of the PAI-1 gene. This places patients with the *PAI-1* 4G/5G genotype in a hypercoagulable state, which is manifested as a thrombotic tendency (10). Although APVT is less common than chronic portal vein thrombosis in cirrhosis, over half of patients have at least one identifiable risk factor; local factors are present in about one-fifth, while approximately one-quarter have no discernible cause (“idiopathic”). Case 2 in our study is consistent with this clinical pattern—after systematic evaluation, no acquired, local, or hereditary risk factors were detected, and it was therefore classified as idiopathic APVT. Notably, SARS-CoV-2 infection impacts all components of Virchow's triad, making venous thromboembolism (VTE), including splanchnic vein thrombosis, a recognized complication of COVID-19, with reported prevalence up to 8% in hospitalized patients (11). Thromboembolic events like APVT can occur weeks after resolution of acute COVID-19 symptoms and have also been associated with COVID-19 vaccination; several cases of APVT linked to SARS-CoV-2 infection have been documented (12, 13). Therefore, in the context of the ongoing pandemic, obtaining relevant SARS-CoV-2 exposure and vaccination history is crucial.

Non-specific abdominal pain is the predominant symptom, occurring in up to 90% of APVT patients, often accompanied by nausea, fever, or diarrhea. Thrombosis extending into mesenteric veins can precipitate intestinal ischemia, manifesting as vomiting, diarrhea, rectal bleeding, or splenomegaly, potentially progressing to sepsis. Untreated vascular occlusion risks catastrophic complications like intestinal perforation (14), peritonitis, shock, and multi-organ failure (2); mortality associated with bowel necrosis can reach 50%–75% (15). Elevated C-reactive protein (CRP) is observed in approximately 80% of patients (16). Based on thrombus extent and vascular occlusion severity, APVT can be classified (6): Grade 1 (portal vein involvement only, occlusion <50%, minimal/no SMV involvement); Grade 2 (portal vein occlusion ≥50%); Grade 3 (complete occlusion of portal vein and proximal SMV); Grade 4 (complete occlusion of portal vein and SMV). Systemic anticoagulation is the first-line treatment (8). Endovascular interventions (mechanical thrombectomy—balloon, rheolytic, or aspiration—and catheter-directed thrombolysis) (17) are indicated for patients with signs of impending bowel ischemia or contraindications to anticoagulation.

### 3.2 CT manifestations and significance

Contrast-enhanced CT is highly accurate, reliable, sensitive, and specific for diagnosing APVT (18). Key direct findings include: venous dilation secondary to acute thrombus; iso- or hyperdense thrombus on unenhanced scans (varying with thrombus composition) (19); perivascular fat stranding due to edema/inflammation (20); and non-enhancing filling defects within the opacified venous lumen on contrast-enhanced scans. Peripheral rim enhancement of the vein may sometimes be seen, attributed to perithrombotic flow or venous wall inflammation (21). CT precisely delineates thrombus location and extent (22). APVT can be categorized based on involved vessels (23): intrahepatic portal vein, extrahepatic portal vein, combined intra/extrahepatic portal vein, or mesenteric/splenic vein. Indirect signs vary: intrahepatic involvement often causes perfusion abnormalities (e.g., transient hepatic attenuation differences); mesenteric vein involvement typically manifests as bowel wall thickening, edema, and luminal dilation, potentially progressing to hemorrhage in severe cases. Other indirect signs include ascites, mesenteric edema/stranding, luminal fluid, and (rarely) portal venous gas or bowel wall pneumatosis. CT also aids in identifying potential underlying causes (e.g., inflammation, malignancy) and complications (e.g., bowel ischemia, portal hypertension features like ascites, splenomegaly, collaterals). Untreated APVT may progress to cavernous transformation of the portal vein, observable within 15–30 days of symptom onset; linear thrombus calcification and established collaterals suggest chronicity (19).

CT is invaluable for post-treatment follow-up. Attali et al. (24) classified outcomes on follow-up CT (within the first year of anticoagulation) as: “Recanalization” (no residual thrombus), “Intermediate” (stable or partially recanalized thrombus), or “Worsening” (increased/extended or new thrombus). Their cohort showed 30%, 52%, and 18% in these groups, respectively. Standardized imaging reporting is essential. The American Association for the Study of Liver Diseases (AASLD) recommends descriptions include (25): Thrombus site/extent: Specify involvement of intrahepatic portal veins, main portal vein trunk, splenic vein, mesenteric veins; describe thrombus length and segmental relationships. Luminal obstruction: Characterize as complete (100% occlusion), partial (>50% occlusion), mild (<50% occlusion), or cavernous transformation. Chronicity/Dynamics: Note chronic features or changes from prior imaging.

### 3.3 Application and value of other imaging modalities in the diagnosis of APVT

Beyond contrast-enhanced computed tomography, ultrasonography and MRI also serve as crucial imaging tools for the diagnosis and follow-up of APVT. In APVT, two-dimensional ultrasonography can demonstrate dilation of the portal vein lumen. The thrombus itself typically appears isoechoic or hypoechoic. Some fresh thrombi, due to a higher red blood cell content, may present as slightly hyperechoic, creating a marked contrast against the surrounding anechoic blood (26). If the thrombus extends to the superior mesenteric or splenic veins, dilation of these respective veins with heterogeneous intraluminal echogenicity can be observed. Color Doppler Ultrasonography (CDU) further enables the assessment of portal venous hemodynamic changes. In complete APVT, the portal vein lumen shows an absence of color flow signals, and pulsed-wave Doppler fails to detect a flow spectrum. Conversely, incomplete APVT is characterized by color flow filling defects within the lumen, accompanied by decreased flow velocity and potentially turbulent signals (18). It is important to note that the diagnostic accuracy of CDU can be influenced by operator expertise, patient body habitus (e.g., obesity), and interference from bowel gas (27).

MRI offers the advantages of being radiation-free and utilizing multi-parametric imaging, making it particularly suitable for pregnant women, pediatric patients, and individuals with contraindications to iodinated contrast media. The MRI appearance of APVT has characteristic features. On T1-weighted imaging (T1WI), acute thrombus (<30 days), rich in deoxyhemoglobin, usually appears isointense or slightly hypointense. On T2-weighted imaging (T2WI), it typically presents as hyperintense; this signal can be more pronounced in fresh thrombi due to their high water content. Magnetic Resonance Venography (MRV), a non-invasive vascular imaging technique, can directly visualize filling defects within the portal vein lumen, clearly defining the extent of thrombus involvement (21).

## 4 Conclusion

APVT is a clinically rare but potentially catastrophic thrombotic disorder. Early diagnosis and appropriate intervention are paramount to prevent adverse outcomes and reduce mortality. Abdominal pain is the most common presenting symptom. Thrombophilia, systemic conditions, and local inflammatory factors are key risk factors; SARS-CoV-2 infection and vaccination are significant clinical considerations. Contrast-enhanced CT is the cornerstone imaging modality, providing critical information for APVT classification, severity assessment, treatment planning and monitoring, and complication detection.

## Data Availability

The original contributions presented in the study are included in the article/Supplementary Material, further inquiries can be directed to the corresponding authors.
